# Extracellular vesicles-released parathyroid hormone-related protein from Lewis lung carcinoma induces lipolysis and adipose tissue browning in cancer cachexia

**DOI:** 10.1038/s41419-020-03382-0

**Published:** 2021-01-28

**Authors:** Wenjun Hu, Hairong Xiong, Zeyuan Ru, Yan Zhao, Yali Zhou, Kairu Xie, Wen Xiao, Zhiyong Xiong, Cheng Wang, Changfei Yuan, Jian Shi, Quansheng Du, Xiaoping Zhang, Hongmei Yang

**Affiliations:** 1 Department of Pathogenic Biology, School of Basic Medicine, Tongji Medical College, Huazhong University of Science and Technology, Wuhan, 430030 Hubei Province China; 2 Department of Urology, Union Hospital, Tongji Medical College, Huazhong University of Science and Technology, Wuhan, 430022 Hubei Province China; 3 Department of Neuroscience and Regenerative Medicine, Medical College of Georgia, Augusta University, Augusta, GA 30912 USA

**Keywords:** Oncogenes, Mechanisms of disease

## Abstract

Cancer cachexia is a metabolic disorder characterized by skeletal muscle wasting and white adipose tissue browning. Specific functions of several hormones, growth factors, and cytokines derived from tumors can trigger cachexia. Moreover, adipose tissue lipolysis might explain weight loss that occurs owing to cachexia. Extracellular vesicles (EVs) are involved in intercellular communication. However, whether EVs participate in lipolysis induced by cancer cachexia has not been thoroughly investigated. Using Lewis lung carcinoma (LLC) cell culture, we tested whether LLC cell-derived EVs can induce lipolysis in 3T3-L1 adipocytes. EVs derived from LLC cells were isolated and characterized biochemically and biophysically. Western blotting and glycerol assay were used to study lipolysis. LLC cell-derived EVs induced lipolysis in vivo and vitro. EVs fused directly with target 3T3-L1 adipocytes and transferred parathyroid hormone-related protein (PTHrP), activating the PKA signaling pathway in 3T3-L1 adipocytes. Blocking PTHrP activity in LLC-EVs using a neutralizing antibody and by knocking down PTHR expression prevented lipolysis in adipocytes. Inhibiting the PKA signaling pathway also prevents the lipolytic effects of EVs. In vivo, suppression of LLC-EVs release by knocking down Rab27A alleviated white adipose tissue browning and lipolysis. Our data showed that LLC cell-derived EVs induced adipocyte lipolysis via the extracellular PTHrP-mediated PKA pathway. Our data demonstrate that LLC-EVs induce lipolysis in vitro and vivo by delivering PTHrP, which interacts with PTHR. The lipolytic effect of LLC-EVs was abrogated by PTHR knockdown and treatment with a neutralizing anti-PTHrP antibody. Together, these data show that LLC-EV-induced lipolysis is mediated by extracellular PTHrP. These findings suggest a novel mechanism of lipid droplet loss and identify a potential therapeutic strategy for cancer cachexia.

## Introduction

Cancer cachexia, a metabolic disorder, is characterized by skeletal muscle wasting and white adipose tissue (WAT) browning^1^. Patients with cachexia may lower their food intake, while exhibiting negative energy balance as a result of hypermetabolism, which cannot be improved by nutritional support^2^. This, in turn, might lead to muscle wasting, which is directly correlated with mortality and a lower quality of life; loss of fat mass is also a prognostic marker for poor outcome^3,4^. Although the mechanisms underlying muscle atrophy have been studied widely, much less is known about the factors that initiate the loss of fat in patients with cancer.

Adipose tissues are classified as WAT and brown adipose tissue (BAT)^5^. They often perform opposite physiological functions, with WAT typically contributing to energy accumulation, and BAT contributing to the dissipation of energy as heat. Fat loss is more rapid than lean tissue loss in cancer cachexia. Interestingly, WAT can switch to BAT, and this phenomenon is called ‘white adipose browning’, which can initiate thermogenesis and induce fat wasting in mice with cancer cachexia^5,6^. Clinical studies suggest that fat depletion in cachexia occurs via lipolysis^5,7,8^. However, the molecular basis of cancer-induced loss of adipose tissue is poorly understood.

Changes in lipid catabolism and lipogenesis drive severe lipid loss in cancer-associated cachexia^7,9^. In particular, glycerol and free fatty acids (FFAs) generated due to hydrolysis of triglycerides, are released into circulation. Indeed, higher levels of FFAs and glycerol are present in the circulation of cachectic patients^7,10^. Both hormone-sensitive lipase (HSL) activity and plasma glycerol are increased in patients with cancer cachexia^7,10,11^. Therefore, we sought to determine the mechanisms underlying fat loss in cancer cachexia. Our study was based on a novel hypothesis that the effects of the tumor on adipose tissue are mediated by its released extracellular vesicles (EVs).

EVs are produced by many cell types and can transfer cell contents (e.g. proteins, microRNAs, mRNAs, lipids, or DNA) among different cells. There are three classes of EVs: microvesicles (100–1000 nm in diameter), apoptotic blebs, and exosomes (30–150 nm). EVs can modulate the biological function of recipient cells by carrying their contents to the cytosol of recipient cells and are key players in tumor progression and drug resistance^12–14^. MicroRNA profiling of circulating tumor exosomes could potentially be used as a surrogate diagnostic procedure and as a noninvasive biomarker for early non-small cell lung cancer diagnosis^15,16^. Exosomes from melanoma cells modify bone marrow progenitor cells towards a pro-metastatic phenotype via mesenchymal–epithelial transition^17^. Moreover, adrenomedullin containing exosomes secreted by pancreatic cancer cells promote lipolysis in adipose tissue^18^, while tumors can induce muscle atrophy in mice by releasing extracellular heat shock proteins (Hsp70 and Hsp90)^19^. Therefore, we hypothesized that tumor-promoting EVs may play a role in the crosstalk between tumor cells and adipocytes, which leads to lipolysis and energy expenditure, and subsequently to fat loss in cancer cachexia.

Parathyroid hormone-related protein (PTHrP) can stimulate thermogenic gene expression by binding to parathyroid hormone receptor (PTHR, which is known to be highly expressed in kidneys, bone, adipose, and muscle tissues^20^), which is shared by parathyroid hormone (PTH) and PTHrP^20^. PTHrP induces lipolysis via protein kinase A (PKA)-mediated phosphorylation of HSL^20,21^. Additionally, bone-derived PTHrP and adiponectin are involved in whole-body metabolism, which is regulated by bone–adipose hormonal relay^22^. Moreover, PTHrP derived from Lewis lung carcinoma (LLC) tumors triggers adipose tissue browning with increased energy production via activation of uncoupling protein 1 (UCP1) and cancer cachexia via binding to PTHR. Interestingly, neutralizing antibodies of PTHrP restrains browning, the loss of adipose depots, and muscle wasting in models of cancer cachexia^21^. Moreover, PTHrP can inhibit adipocyte differentiation by downregulating PPARγ activity via a MAPK-dependent mechanism^23^. Additionally, PTHrP can be secreted by numerous tumors and is related to hypercalcemia of malignancy associated with cancer cachexia^24–26^. Therefore, we proposed that PTHrP in EVs could serve as a mediator of lipolysis and browning in cancer cachexia.

The conditioned medium from LLC cells (LCM) activates a catabolic response in cultured 3T3-L1 adipocytes that demonstrate lipids metabolism in LLC tumor-bearing mice^21,27^. This finding suggests that LLC cells release cachexins that directly induce lipolysis and adipose tissue browning. Additionally, we previously found that cachexia-inducing tumor cells release high levels of EVs that serve as carriers of tumor-released PTHrP. However, the role of EVs in lipolysis and the effectors utilized in the process are as of yet unknown.

In the present study, we found that lipolysis occurs in adipocytes upon exposure to LLC-derived EVs (LLC-EVs). To confirm the role of PTHrP, we blocked PTHrP activity in LLC-EVs by using a neutralizing antibody. Furthermore, we assessed WAT browning and fat loss in mice implanted with LLC cells in which the expression of Rab27A (a GTPase that controls different steps of EV release) had been stably knocked down with shRNA.

## Materials and methods

### Reagents

Recombinant mouse PTHrP was purchased from Wuhan USCN Business Co. Ltd. (Wuhan, China). We purchased anti-PTHrP (1-34) antibody (T-4512) from Peninsula Laboratories International (San Carlos, CA, USA). H89 (ab143787), the inhibitor of PKA, and anti-UCP1 (ab10983) were purchased from Abcam (Cambridge, MA, USA). All other antibodies were purchased from Cell Signaling Technology (Danvers, MA, USA).

### LLC cell culture and animals

LLC cells were maintained in Dulbecco’s modified Eagle’s medium (DMEM) (Invitrogen, Carlsbad, CA, USA) with 10% fetal bovine serum, 1% penicillin, and streptomycin. C57BL/6 mice from Beijing HFK Bioscience (Beijing, China) were used for all experiments. We used 6- to -10-week-old male mice in all experiments. The mice were randomly divided into four groups (each group has nine mice, *n* = 9): mice injected with PBS, mice injected with LLC cells, mice injected with LLC cells transduced with lentiviral shRab27A, and mice injected with LLC cells transduced with lentiviral control. Cells (5 × 10^6^) were injected subcutaneously into the flanks of the mice. Mice were euthanized on day 21 following tumor implantation. Body weight was then determined and iWAT and eWAT were immediately harvested and weighed. For subsequent studies, the iWAT and eWAT were fixed in 4% paraformaldehyde and the other tissues were immediately frozen in liquid nitrogen and stored at −80 °C.

### Lipid droplet areas

We chose 3 representative images of each section from three individual mice to quantify the lipid droplet areas using ImageJ software (National Institutes of Health, Bethesda, MD). The detailed protocol has been described previously^27^.

### Isolation of LLC-EVs

EV extraction was performed according to previously published protocols^18^. Briefly, cells were grown to ∼70% confluence and cultured in 10% exosome-free fetal bovine serum (System Biosciences, Palo Alto, CA, USA) for 72 h. We collected the culture medium, which we subjected to centrifugation at 3000 rpm for 10 min at 4 °C to remove debris. We then subjected the supernatant to centrifugation at 100,000 × *g* for 60 min using an ultracentrifuge (Beckman, Brea, CA, USA) to pellet the EVs. EVs were then washed in PBS and subjected to centrifugation at 100,000 × *g* for 60 min. The resulting pellets were resuspended in PBS. We assessed the protein concentrations in the EVs with a BCA Protein Assay Kit (Thermo Fisher, Waltham, MA, USA).

### 3T3-L1 cell culture and differentiation

Murine 3T3-L1 cells were obtained from the American Type Culture Collection (Manassas, VA, USA) and cultured in DMEM with 10% fetal calf serum. Confluent 3T3-L1 cells were treated with a cocktail of 0.5 mM isobutylmethylxanthine, 1 mM dexamethasone, 5 mg/ml insulin, and 5 mM troglitazone (all from Sigma–Aldrich, St Louis, MO, USA) to induce differentiation. After 2 days of differentiation, the cells were maintained in a medium with insulin until harvest. Experiments were conducted using differentiated adipocytes (10–14 days).

### Oil red O staining of adipocytes

Adipocytes were stained with Oil red O solution (Goodbio Technology, Wuhan, China). Briefly, cells were washed three times with PBS and fixed in 4% formalin. After fixation, we diluted 0.5% Oil red O in isopropanol (Sigma-Aldrich) with water (3:2), filtered the solution through a 0.45-µm filter, and incubated with the fixed cells for 1 h at room temperature. After staining, images were obtained using a Leica DMI 3000B microscope (Wetzlar, Germany).

### Glycerol estimation assay

Differentiated adipocytes were serum-starved overnight in DMEM and 2% bovine serum albumin (fatty acid-free,) then treated with exosome-free DMEM (phenol red-free) with 0.5% bovine serum albumin for 24 h. We assayed the supernatants for glycerol levels with free glycerol reagent (Sigma–Aldrich), as per the manufacturer’s recommendations.

### Internalization of labeled EVs

EVs were labeled with 2 μM PKH67 (Sigma–Aldrich) for 4 min, washed, then incubated for 12 h with cultured 3T3-L1 adipocytes (∼3000 cells/well). Cells were washed, counterstained with 2-(4-amidinophenyl)-1H-indole-6-carboxamidine, and imaged under an Olympus FV500 fluorescence microscope (Tokyo, Japan).

### Electron microscopy

EVs were fixed in 1% glutaraldehyde in PBS (pH 7.4). After rinsing, a 30-μl drop of the suspension was loaded onto a formvar/carbon-coated grid, negatively stained with 3% (w/v) aqueous phosphotungstic acid for 90 s, and observed by transmission electron microscopy (JEM 1200EX, Jeol, Tokyo, Japan).

### Nanoparticle analysis

We analyzed the EV fractions on a Malvern instrument (Malvern Instruments, Malvern, UK). The EV particles were counted and analyzed using the Nanoparticle Tracking Analysis software.

### Western blotting

Western blotting was performed as previously described^27^. Briefly, proteins from adipose tissue, differentiated 3T3-L1 cells, and EVs were extracted using radioimmunoprecipitation assay protein lysis buffer (P1003; Beyotime Institute of Biotechnology, Shanghai, China) with freshly added 1% protease inhibitor cocktail and 1 mM phenylmethylsulphonyl fluoride. Each well was loaded with 50 μg protein and incubated overnight at 4 °C with primary antibodies. We purchased antibodies phospho-PKA substrate (catalog number 9624), phospho-HSL (catalog number 4126), and HSL (catalog number 4107) from Cell Signaling Technology; antibodies against UCP1(catalog number ab10983), tubulin (catalog number ab18207), CD9 (catalog number ab92726), TSG101 (catalog number 125011), and Hsp70 (catalog number 2787) from Abcam; and an antibody against Rab27A from Proteintech (Wuhan, China). We performed quantitative analyses of protein levels with ImageJ software.

### Immunofluorescent staining

3T3-L1 adipocytes were seeded and differentiated on chamber slides. The cells were washed with PBS, fixed in 4% paraformaldehyde, permeabilized with 0.3% Triton^™^ X-100 for 15 min, and blocked with 3% bovine serum albumin for 30 min. Cells were then incubated with antibodies against PTHrP and PTHR, followed by Alexa Fluor^®^ 488- or Alexa Fluor^®^ 594-conjugated secondary antibody. Nuclei were stained with 2-(4-amidinophenyl)-1H-indole-6-carboxamidine. Images were taken with an Olympus FV500 fluorescence microscope (Tokyo, Japan).

### shRNA expression in LLC and 3T3-L1 cells

For the shRNA-mediated knockdown of Rab27A and PTHR, we purchased lentiviral vectors encoding shRNAs from GeneChem (Shanghai, China) and packaged them in viral particles according to the manufacturer’s recommendations. Mouse Rab27A shRNA sense sequences used were:5’- AGTACACTGATGGCAAGTT-3’; PTHR sense sequences used were:5’-CCAGCCATTGAGAACGA-AA-3’; LLC cells transduced with shRNA targeting Rab27A and 3T3-L1 preadipocytes transduced with shRNA targeting PTHR were selected by puromycin.

### Statistical analyses

Data are presented as the mean ± standard error of the mean. We performed one-way analysis of variance followed by Bonferroni’s post hoc correction to compare the conditions in both cell and animal experiments. Differences were deemed significant when the *p* value was <0.05, (*P < 0.05, **P < 0.01, ***P < 0.001, ****P < 0.0001).

## Results

### Characterization of LLC-EVs

**Fig. 1:**
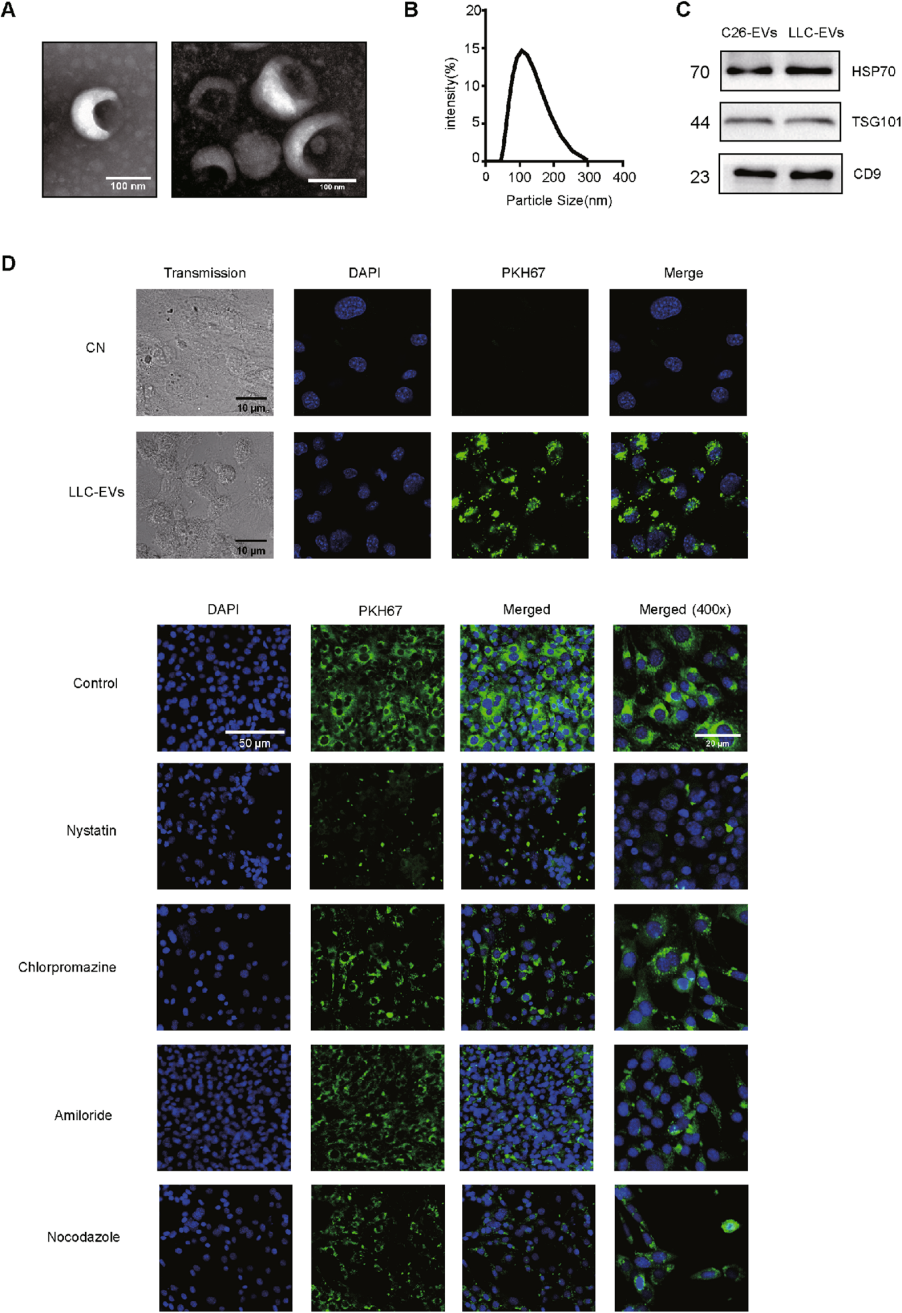
Analysis of LLC-EV characteristics. **A** Analysis of EVs by electron microscopy (bars = 100 nm). **B** Size of LLC-EVs isolated from LLC cells (mean diameter: 109 nm). **C** Western blotting analysis of the expression of Hsp70, TSG101, and CD9 in EVs isolated from C26 and LLC cells. **D** Confocal images (20×) of differentiated adipocytes pretreated with various inhibitors of endocytic pathways and incubated with labeled LLC-EVs for overnight at 37 °C. Amiloride (50 μM; 30 min), nystatin (54 μM; 30 min), chlorpromazine (20 μM; 30 min), and nocodazole (1 μM; 10 min).

We isolated LLC-EVs from serum-free culture medium through multistep centrifugation^18^. Analysis of LLC-EVs by electron microscopy revealed the cup-shaped vesicles (Fig. 1A). We quantitatively characterized the mean LLC-EV diameter as 109 nm using a Zetasizer Nano (Fig. 1B). Furthermore, we detected the exosomal markers Hsp70, TSG101, and CD9 in LLC-EVs and colon-26 (C26)-derived EVs (C26-EVs; C26 that also induce cachexia in mice) by western blotting analysis (Fig. 1C). Then, we labeled the EVs with PKH67, incubated with 3T3-L1 adipocytes, and observed green fluorescence in the adipocytes by confocal fluorescence microscopy (Fig. 1D). However, the internalization of LLC-EVs into adipocytes could be inhibited by nystatin (54 μM, an inhibitor of caveolin/lipid raft-mediated endocytosis); While, chlorpromazine (20 μM, an inhibitor of clathrin-mediated endocytic pathway), amiloride (50 μM, an inhibitor of micropinocytosis), and nocodazole (1 μM, a destabilizer of microtubule) did not exhibit present the analogous effect. This finding suggested that adipocytes take up EVs through caveolin/lipid raft-mediated endocytosis.

### LLC-EVs induced lipolysis in vitro

**Fig. 2:**
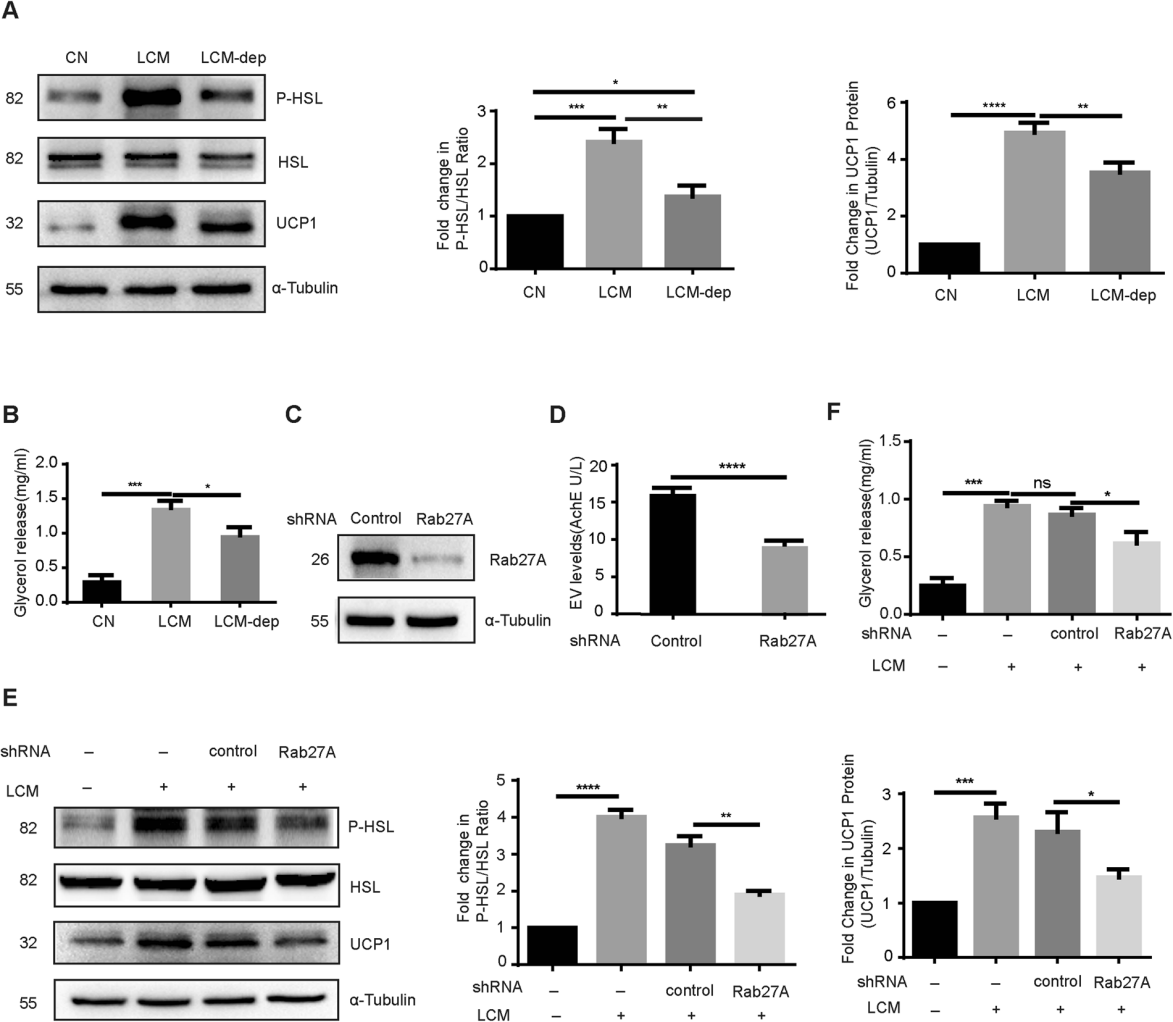
LLC-EVs have a lipolytic action in 3T3-L1 adipocytes. **A** Western blotting showing the relative level of phospho-HSL: HSL and UCP1 in 3T3-L1 adipocytes. Data (*n* = 3) were analyzed by analysis of variance (ANOVA). **B** Glycerol levels in the supernatants of 3T3-L1 adipocytes incubated with LCM or LCM-dep. Data (*n* = 3) were analyzed by analysis of variance (ANOVA). **C** Western blotting showing the level of Rab27A in LLC cells. **D** AchE levels in supernatants of LLC cell transfected with shRNA-Rab27A or control lentivirus vector. Data (*n* = 4) were analyzed by analysis of variance (ANOVA). **E** Western blotting showing the relative level of phospho-HSL: HSL and UCP1 in 3T3-L1 adipocytes incubated with or without LCM, and LCM (Rab27A shRNA or control shRNA). Data (*n* = 8) were analyzed by analysis of variance (ANOVA). **F** Glycerol levels in the supernatants of 3T3-L1 adipocytes incubated with or without LCM, and LCM (Rab27A shRNA or control shRNA). Data (*n* = 3) were analyzed by analysis of variance (ANOVA). *P < 0.05, **P < 0.01, ***P < 0.001, ****P < 0.0001, ns: not significant.

To explore the effect of LLC-EVs on lipid metabolism, we incubated 3T3-L1 adipocytes with LCM (LLC-cell-conditioned medium), which contains EVs, and EV-depleted LCM (LCM-dep). We measured lipolysis by assessing glycerol release and by western blotting for phosphorylated HSL (a marker of lipolysis) and the level of UCP1 (uncoupling protein 1; a mitochondrial protein expressed in multilocular cells that can uncouple oxidative phosphorylation and produce heat). Western blotting of 3T3-L1 adipocytes demonstrated that LCM activated lipolysis, whereas the effect of LCM-dep was comparatively weak (Fig. 2A). In the glycerol assay, the degree of glycerol release was higher in response to LCM than LCM-dep (Fig. 2B). These findings suggested that LLC-EVs may be involved in lipolysis.

To further explore the role of LLC-EVs in lipolysis, we disrupted tumor cell release of EVs by knocking down the expression of Rab27A^28,29^ (Fig. 2C). Subsequently, we quantified the EVs in the supernatants of LLC cell cultures by assessing the activity of acetylcholinesterase (AchE)^30^. Treatment with LCM from cells exposed to Rab27A shRNA inhibited the activity of AchE compared to treatment with the LCM from cells treated with the control lentiviral vector (Fig. 2D). Furthermore, western blotting suggested that the ratio of phospho-HSL: HSL and the expression of UCP1 were lower in adipocytes incubated with LCM from cells exposed to Rab27A shRNA than in those treated with the control LCM (Fig. 2E). In addition, glycerol release from adipocytes incubated with LCM from cells exposed to Rab27A shRNA was lower than from those treated with control LCM (Fig. 2F). These findings further confirmed that LLC-released EVs contribute to lipolysis.

### PTHrP contained in LLC-EVs was involved in lipolysis in 3T3-L1 adipocytes

**Fig. 3:**
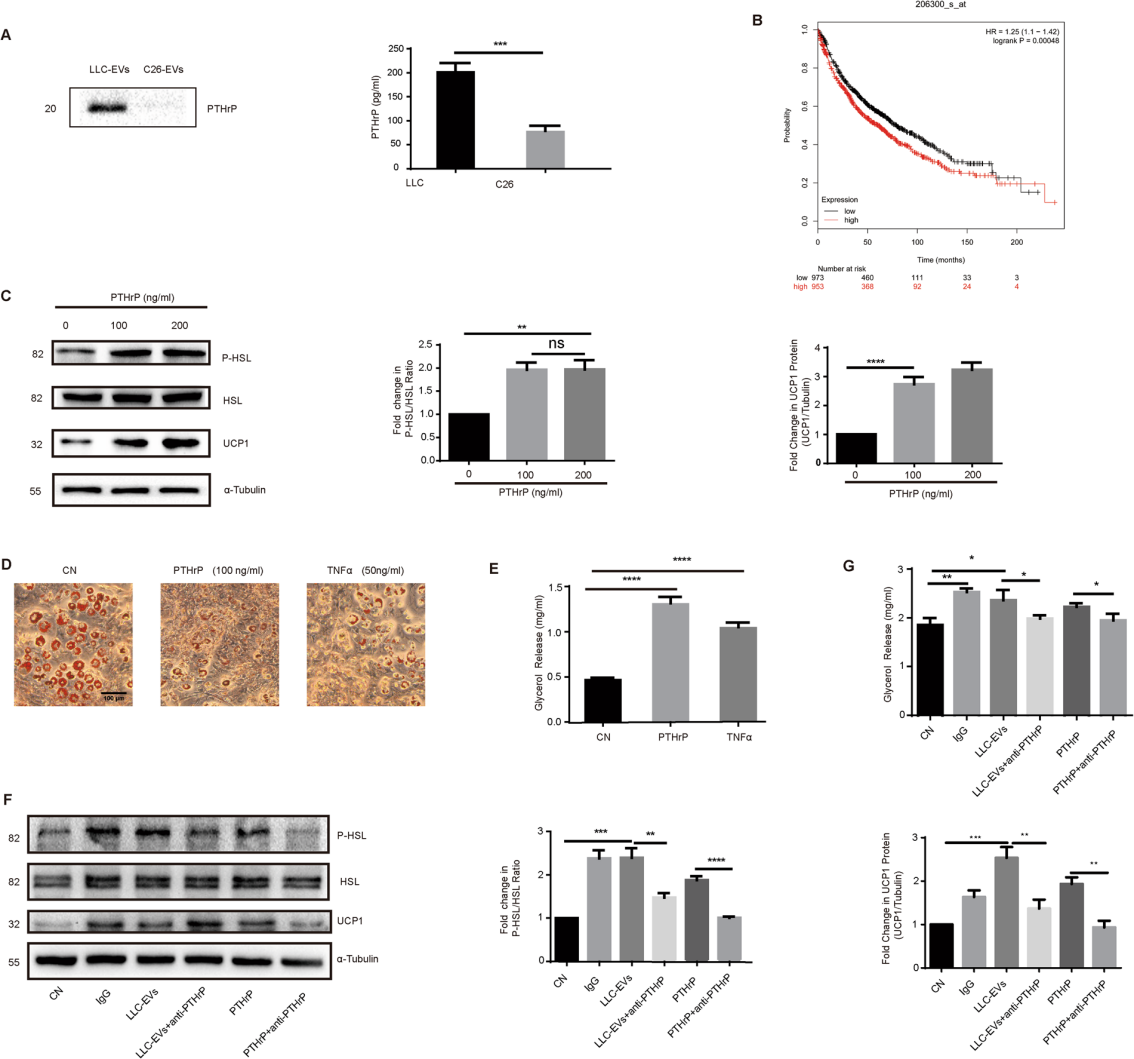
PTHrP in LLC-EVs participated in lipolysis in 3T3-L1 adipocytes. **A** Western blotting showing the presence of PTHrP in LLC-EVs (50 μg) but not clearly in C26-EVs (50 μg) (there is no appropriate internal control in extracellular vesicles. Therefore, we detected the expression of UCP1 from the same quantity of protein sample loaded); The concentrations of PTHrP in the supernatants from LLC and C26 cultures. Data (*n* = 4) were analyzed by Student *t* test. **B** High expression of PTHrP in a tumor predicts a worse overall survival for patients with lung cancer. **C** Western blotting showing the relative levels of phospho-HSL: HSL and UCP1 in adipocytes treated with different doses of PTHrP (100 ng/ml and 200 ng/ml). Data (*n* = 7) were analyzed by analysis of variance (ANOVA). **D** Oil red O staining of 3T3-L1 adipocytes cultured with PTHrP (100 ng/ml) or TNF-α (50 ng/ml). Bar = 100 µm. **E** The levels of glycerol release in 3T3-L1 adipocytes treated with LLC-EVs(10 μg) or TNF-α for 24 h. Data (*n* = 3) were analyzed by analysis of variance (ANOVA). **F** Western blotting showing the relative levels of phospho-HSL and HSL in 3T3-L1 adipocytes treated with PTHrP (with and without anti-PTHrP) and with LLC-EVs (with and without anti-PTHrP). Data (*n* = 3) were analyzed by analysis of variance (ANOVA). **G** The levels of glycerol release from 3T3-L1 adipocytes treated with PTHrP (with and without anti-PTHrP) and with LLC-EVs (with and without anti-PTHrP) for 24 h. Data (*n* = 4) were analyzed by analysis of variance (ANOVA). *P < 0.05, **P < 0.01, ***P < 0.001, ****P < 0.0001, ns: not significant.

PTHrP released by LLC cells into LCM promotes thermogenic gene expression in adipocytes. Interestingly, we observed that PTHrP can be detected in LLC-EVs obviously, but not clearly in C26-EVs. Furthermore. Subsequently, we assessed the levels of PTHrP in the supernatant of various tumor cells capable of triggering cachexia, including mouse lung carcinoma cell (LLC) and C26 colon carcinoma cells (C26). We detected higher levels of PTHrP in the supernatant of LLC than in that of C26 by enzyme-linked immunosorbent assay (Fig. 3A). Therefore, the constitutive release of PTHrP is not a common characteristic shared by different types of cancer cells that can induce cancer cachexia. Furthermore, utilizing patient data from the database associated with the web-based Kaplan–Meier plotter KmPlot, we divided patients into two groups based on the mRNA expression levels in their tumor biopsies. We observed a significantly shorter overall survival for patients with lung cancer with PTHrP mRNA levels above the median value (hazard ratio = 1.25 [1.1–1.42], logrank *P* = 0.00048, *n* = 2,437; Fig. 3B). To assess the effect of PTHrP on lipid metabolism, we treated 3T3-L1 adipocytes with various doses of PTHrP (100 ng/ml and 200 ng/ml) and immunoblotted to detect their expression of phospho-HSL, HSL, and UCP1. The ratio of phospho-HSL: HSL and level of UCP1 expression were significantly higher in adipocytes treated with PTHrP (Fig. 3C). Additionally, we observed fewer lipid droplets in 3T3-L1 adipocytes treated with PTHrP (100 ng/ml) and higher levels of glycerol in the supernatants after PTHrP treatment compared with those in the control group (Fig. 3D, E); TNF-α was used as a positive control. Next, western blotting demonstrated that the ratio of phospho-HSL: HSL and level of UCP1 expression increased upon treatment with PTHrP and LLC-EVs, whereas treatment with anti-PTHrP antibody neutralized this effect (Fig. 3F). Furthermore, we assessed glycerol release in response to PTHrP in cell supernatants and LLC-EVs in the presence and absence of an anti-PTHrP neutralizing antibody. Notably, the anti-PTHrP antibody alleviated lipolysis induced by PTHrP and LLC-EVs (Fig. 3G). These findings further confirmed that the induction of lipolysis by LLC-EVs is partially dependent on extracellular PTHrP.

### PTHR is critical to LLC tumor-induced lipolysis

**Fig. 4:**
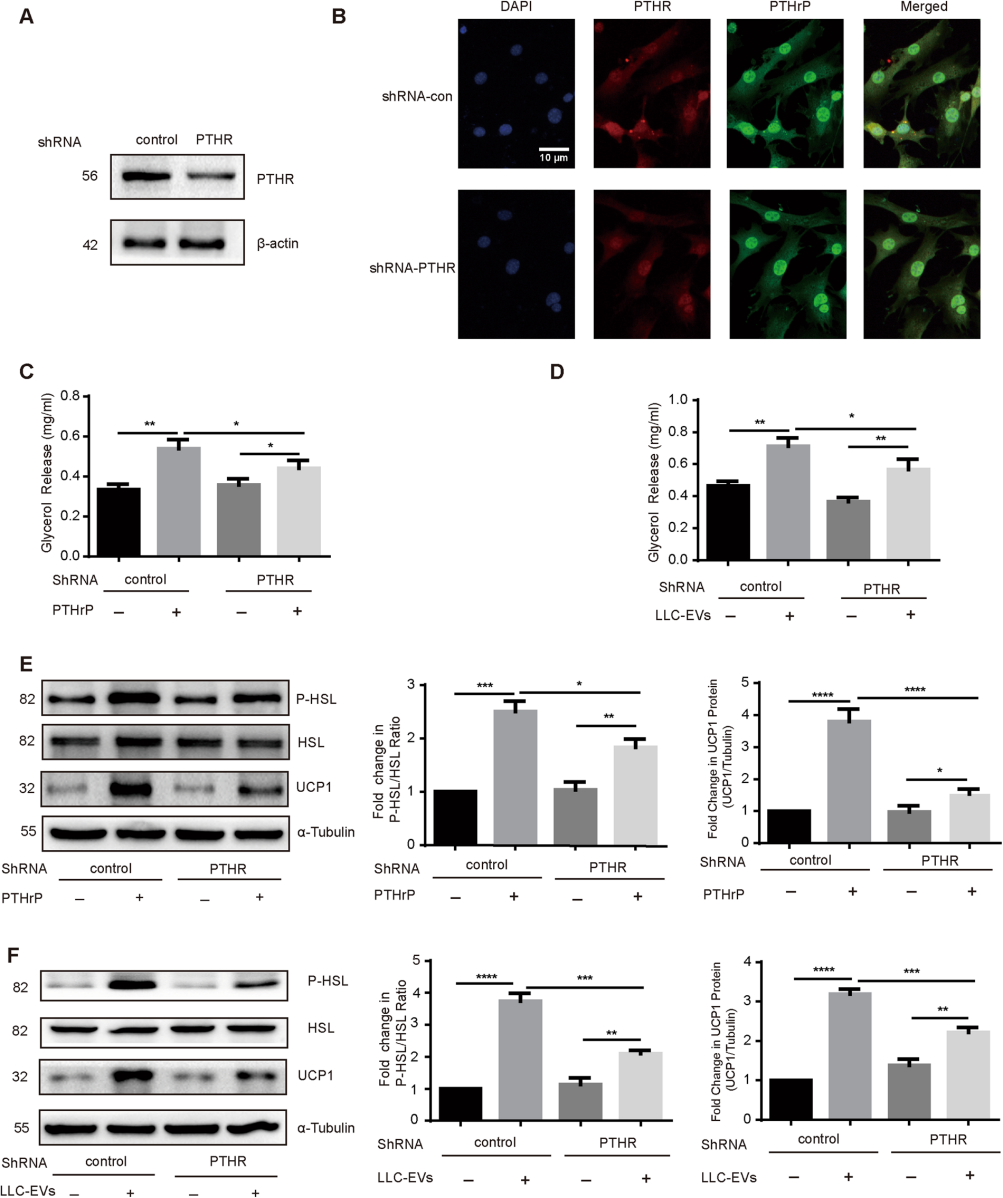
Lipolysis induced by LLC-EVs was partially dependent on extracellular PTHrP. **A** Western blotting analysis of the expression of PTHR. **B** PTHR interactions as assessed by immunofluorescence in 3T3-L1 adipocytes: treated with LLC-EVs (10 μg), and treated with LLC-EVs (10 μg) from cells transduced with the lentivirus encoding PTHR. Bar = 10 μm. **C**, **D** Glycerol release from 3T3-L1 adipocytes transduced with the lentivirus encoding PTHR or untransduced cells in the presence or absence of PTHrP (100 ng/ml) and LLC-EVs (10 μg) for 24 h. Data (*n* = 4) were analyzed by analysis of variance (ANOVA). **E**, **F** Western blotting showing phospho-HSL and HSL in 3T3-L1 adipocytes transduced with the lentivirus encoding PTHR or untransduced cells in the presence or absence of PTHrP (100 ng/ml) and LLC-EVs (10 μg) for 24 h. Data (*n* = 6) were analyzed by analysis of variance (ANOVA). *P < 0.05, **P < 0.01, ***P < 0.001, ****P < 0.0001.

PTHR-mediated wasting in cachexia occurs through a common mechanism involving PTHR. Hence, we investigated if lipolysis was induced by PTHrP and LLC-EVs through interaction with PTHR by transfecting 3T3-L1 adipocytes with shRNA targeting PTHR, PTHR expression had been stably knocked down (Fig. 4A). Confocal localization showed more PTHrP/PTHR interactions, characterized by yellow punctate dots formed by merged red and green punctate dots, in the presence of increasing amounts of LLC-EVs. However, LLC-EVs treatment of adipocytes treated with PTHR shRNA abolished the receptor–ligand interactions (Fig. 4B). The lipolysis induced by PTHrP and LLC-EVs as assessed by glycerol release was also suppressed by treatment with shRNA targeting PTHrP (Fig. 4C, D). Furthermore, we observed that the effects of PTHrP and LLC-EVs on the ratio of phospho-HSL: HSL and UCP1 required PTHR (Fig. 4E, F), suggesting that LLC-EVs and PTHrP induce lipid droplet lipolysis via interaction with PTHR on 3T3-L1 adipocytes. Similarly, thus, our results demonstrate that 3T3-L1 adipocytes lacking PTHR are resistant to lipolysis driven by tumor cell-released EVs.

### Lipolytic effect of extracellular PTHrP was mediated through the PKA pathway

**Fig. 5:**
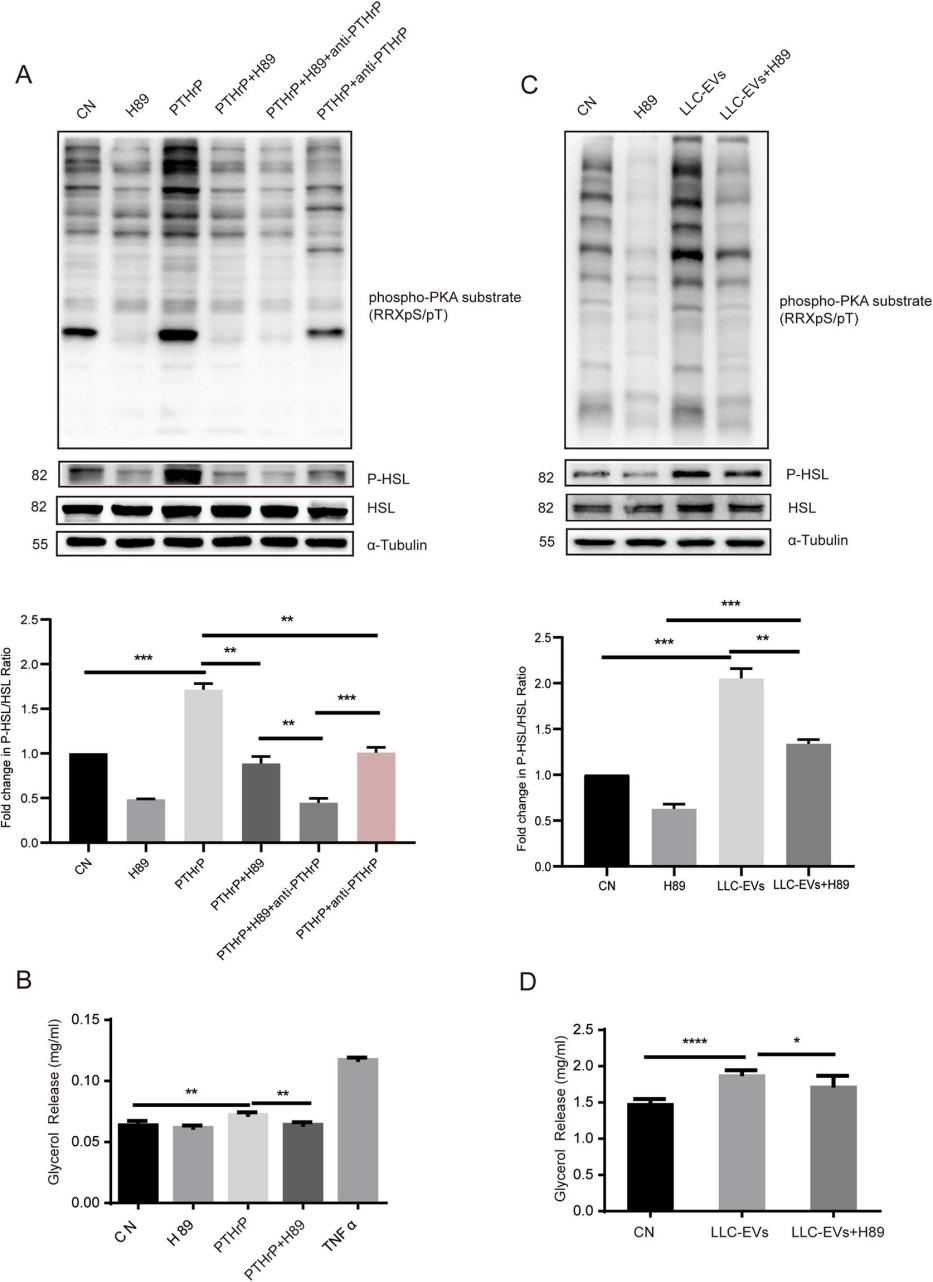
The lipolytic effect of extracellular PTHrP was mediated through the PKA pathway. 3T3-L1 adipocytes were serum-starved for 2 h and pretreated with 50 μM H89 for 1 h, then PTHrP (100 ng/ml) was added for 30 min to analyze protein phosphorylation by western blotting (**A**) or for 24 h to assess glycerol release (**B**). 3T3-L1 adipocytes were serum-starved for 2 h and pretreated with 50 μM H89 for 1 h, then LLC-EVs (10 μg) were added for 30 min to analyze protein phosphorylation by western blotting (**C**) or for 24 h to assess glycerol release (**D**). Data (*n* = 3) were analyzed by analysis of variance (ANOVA). *P < 0.05, **P < 0.01, ***P < 0.001, ****P < 0.0001.

PTHrP has been shown to interact with the G protein-coupled PTHR, which stimulates the phosphorylation of the PKA substrates HSL^20^. Hence, we investigated if extracellular PTHrP-induced lipolysis through the activation of PKA in adipocytes by examining the effects of H89, a selective PKA inhibitor. Western blotting showed that the PKA pathway activation and increased HSL phosphorylation in adipocytes induced by PTHrP were suppressed by H89 (Fig. 5A). Similarly, the PTHrP-induced increase in glycerol release by 3T3-L1 adipocytes was suppressed by H89 (Fig. 5B). Interestingly, western blotting revealed that the effects of LLC-EVs on PKA pathway activation and HSL phosphorylation in adipocytes were suppressed by H89 (Fig. 5C), as was their effect on glycerol release (Fig. 5D). These findings suggested that tumor cells released extracellular PTHrP-induced lipolysis by activating PKA-mediated lipid catabolism.

### Tumor-released PTHrP-expressing EVs caused lipolysis and WAT browning

**Fig. 6:**
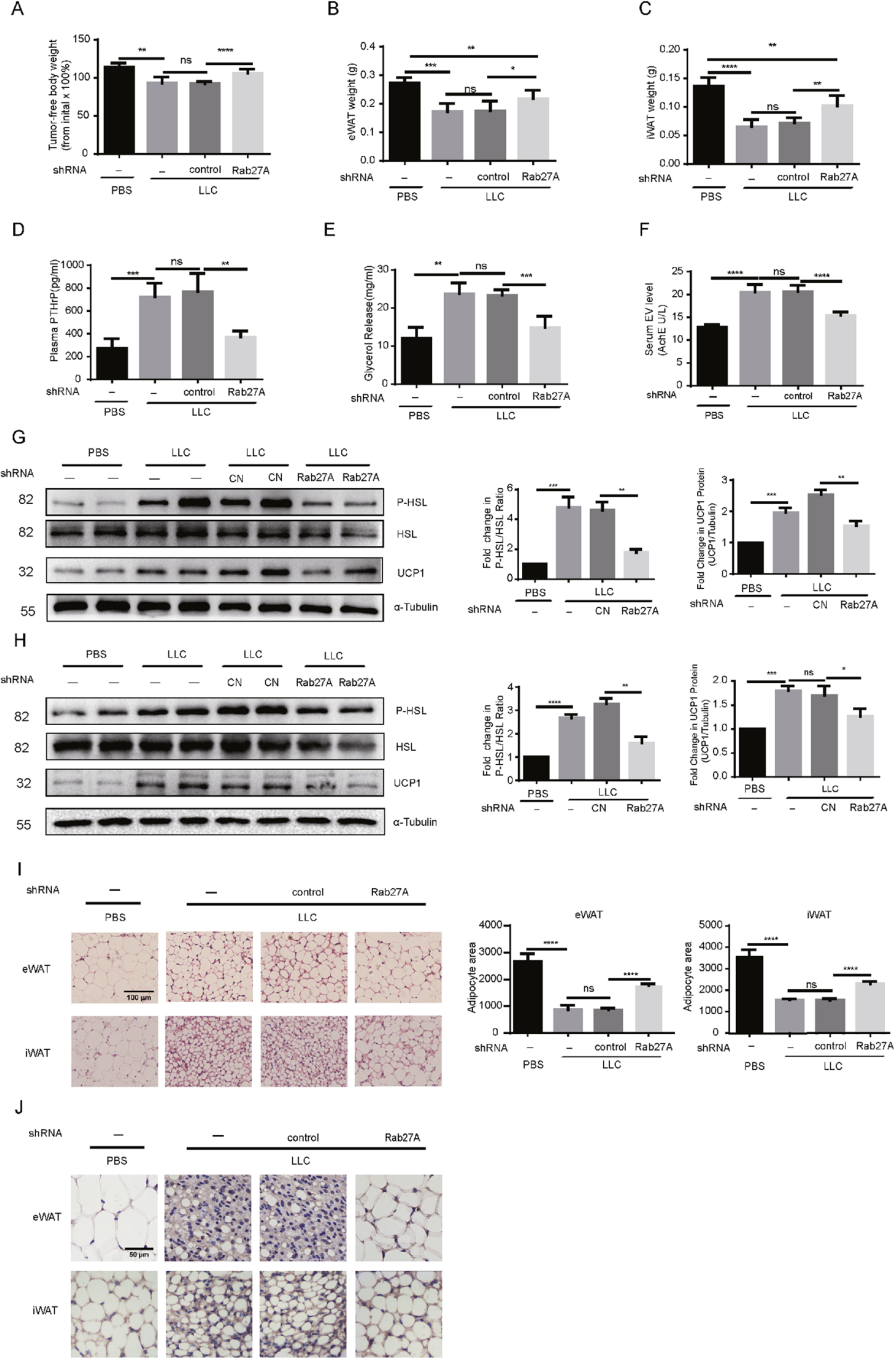
Tumor-released PTHrP-expressing EVs caused lipolysis and WAT browning (*n* = 9 for each group). (**A**) Weight change of the tumor-free body. Data (*n* = 3) were analyzed by analysis of variance (ANOVA) (**B**, **C**) Weight change of iWAT and eWAT. Data (*n* = 3) were analyzed by analysis of variance (ANOVA) (**D**, **E**, **F**) Level of PTHrP concentration, glycerol release, and serum AchE activity in the serum of mice. Data (*n* = 3) were analyzed by analysis of variance (ANOVA) (**G**, **H**) western blotting analysis the level of P-HSL and UCP1 in eWAT and iWAT. Data (*n* = 3) were analyzed by analysis of variance (ANOVA) (**I**, **J**) Hematoxylin and eosin and UCP1 staining of eWAT and iWAT. Data (*n* = 3) were analyzed by analysis of variance (ANOVA). * P < 0.05, ** P < 0.01, *** P < 0.001, ****P < 0.0001, ns: not significant.

To determine whether tumor cell-released PTHrP-expressing EVs are critical for adipose tissue lipolysis and browning, we disrupted LLC release of EVs in vivo by knocking down the expression of Rab27A before tumor implantation. We observed that mice implanted with LLC cells in which Rab27A expression had been stably knocked down did not experience abnormal decreases in free body, inguinal WAT (iWAT), or epididymal WAT (eWAT) weight, or increases in serum glycerol release, serum AchE activity, or PTHrP levels (Fig. 6A–F). Furthermore, western blotting showed that the ratio of phospho-HSL: HSL and the expression of UCP1 were suppressed in eWAT and iWAT of mice implanted with LLC cells lacking Rab27A (Fig. 6G, H). Similarly, hematoxylin and eosin revealed significantly smaller adipocytes with multilocular cytoplasm in both the eWAT and iWAT of mice implanted with LLC cells lacking Rab27A compared to not lacking Rab27A (Fig. 6I); UCP1 staining revealed significantly that the expression of UCP1 is lower in both the eWAT and iWAT of mice implanted with LLC cells lacking Rab27A compared to not lacking Rab27A (Fig. 6J). These findings suggested that targeting tumor cell-released PTHrP-expressing EVs could be an effective therapeutic strategy for cancer cachexia.

## Discussion

Our study provides a novel insight into the function of tumor-derived EVs. We found that LLC-EVs fuse with and promote the lipolysis of adipocytes in vitro and in vivo via activation of PKA signaling. Importantly, our data suggest that tumor-released extracellular PTHrP is a key cachexin responsible for adipose loss in tumor-bearing mice.

PTHrP is secreted by many tumors and has a known role in cancer cachexia. It affects lipolysis and inhibits adipocyte differentiation^20,23^. In our study, we observed that both recombinant and extracellular PTHrP-promoted lipolysis through the activation of the PKA pathway; this effect was abrogated in the presence of an anti-PTHrP antibody. However, in our C26-induced cachexia model, we also observed WAT browning and fat droplet loss in the presence weakly of PTHrP containing C26-EVs. The phenomenon demonstrated that different types of tumor-derived EVs carry different cargoes and may mediate different pathways of internalization into recipient cells^31,32^. This suggests that different tumors induce similar phenotypes via different mechanisms that must be targeted by distinct therapeutic modalities.

Our data demonstrate that LLC-EVs induce lipolysis in vitro, which is abrogated by PTHR knockdown, suggesting that LLC-EV-induced lipolysis is partially dependent on extracellular PTHrP. We also showed that LLC-EVs do not contain functionally relevant levels of TNF-α, a well-known lipolytic factor (Figure S1). We speculate that other factors may contribute to LLC-induced lipolysis either independently or in conjunction with LLC-derived extracellular PTHrP. Additionally, to explore the distribution of tumor-derived EVs in vivo, we injected PKH-67 labeled EVs into the tail veins of mice. In subcutaneous adipose tissue and epididymal adipose tissue, we could observe the presence of labeled EVs (Figure S2). However, the effects of LLC-EVs in vivo and the mechanism behind lipolysis induced by LLC-EVs in vivo and vitro will be further explored in our future studies.

EVs can provide therapeutic opportunities for the treatment of various types of tumors^33^. We detected tumor-derived EVs in supernatants of LLC that were similar to exosomes in terms of size (~109 nm) and protein expression (Hsp70/TSG101/CD9). We used the term ‘extracellular vesicles’ for these vesicles to conform to the recommendations of the International Society for Extracellular Vesicles^34^. Targeting Rab27B can inhibit exosome-mediated transfer miR-34c-5p and increase its intracellular level; this research provides a new strategy for the treatment of patients with acute myeloid leukemia^35^. Inhibiting exosome generation in sepsis by GW4869 can suppress the sepsis triggered inflammatory response and then improve cardiac function and survival^36^. Similarly, inhibiting cancer cell-derived EVs release and biogenesis by GW4869 can change the EV emission profiles reflective of drug-related therapeutic stress and thereby, EV-based assays can serve as companion diagnostics for targeted anticancer agents^37^. Additionally, EVs released by cancer cells can be used as effective carriers of paclitaxel to their parental cells, carrying the drug into cells and increasing its cytotoxicity^38,39^. Furthermore, EVs can be used as therapeutics and as diagnostic biomarkers in clinical application^40,41^. In the cancer cachexia model, inhibiting LLC-EVs release by knocking down the expression of Rab27A and Rab27B can alleviate muscle wasting^19^. In our study, inhibiting LLC-EVs release by knocking down Rab27A expression could relieve fat loss and WAT browning in cancer cachectic mice. However, in patients with cancer, how to reasonably utilize EVs in clinical therapeutics for cachexia still requires more effort and further research.

In our study, we showed that tumor-released EVs mediate lipolysis of lipid droplets through the cargo protein PTHrP. Kri et al. previously reported that LLC tumors released a high level of PTHrP, which induced WAT browning and fat loss^21^. However, Zhang et al. reported that PTHrP levels released into culture supernatants by LLC cells were similar to those released by non-tumorigenic cells and that serum PTHrP levels in mice with cachexia induced by LLC cells were comparable to those in controls^19^. In our study, we observed that serum PTHrP levels are higher in cachectic mice than in control mice. Furthermore, serum PTHrP concentrations in mice bearing LLC transduced with shRNA against Rab27A are lower than those in mice injected with LLC transduced with the empty vector. In addition, we detected PTHrP in EVs released by LLC cells but not in EVs released by C26 cells, which also induced cachexia with WAT browning. Therefore, we conclude that different LLC cell subtypes release distinct cachexins and EVs with different contents.

We observed that recombinant PTHrP and extracellular PTHrP exert their effects by interacting with PTHR (encoded by *Pth1r*) on adipocytes. Previous reports have also demonstrated that PTHrP promotes lipolysis by binding to PTHR and activating the PKA pathway^20,42^. Similarly, we found that PTHrP and LLC-EVs activate the PKA pathway and lipolysis, which can be partially inhibited by H89. This finding further confirmed that LLC-EVs contain PTHrP. Based on these observations, we propose that tumor-released EVs induce WAT browning and lipid droplet loss by activating the PKA/CREB/HSL catabolic signaling pathway. Furthermore, lung tumor exosomes can suppress adipogenesis of human mesenchymal stem cells through TGFβ signaling pathway^43^. Therefore, we hypothesize that the effects of reduced adipogenesis and increased lipolysis induced by tumor-released EVs, may contribute to fat loss and WAT browning in cancer cachexia. This hypothesis and possible mechanisms require further research. By packaging specific cargo, LLC-EVs appear to function as important mediators of paracrine intercellular communication and may involve specific pathways for internalization in recipient cells (including adipocytes), depending on their cargo, size, and cell signaling status^31^. In our study, we observed caveolin/lipid raft-mediated endocytosis as a possible mechanism for EVs internalization in adipocytes. However, other mechanisms and molecules mediating EVs internalization into recipient cells still require exploration to provide a strategy for clinical therapy.

Our data demonstrate that LLC-EVs induce lipolysis in vitro and in vivo by delivering PTHrP, which interacts with PTHR. The lipolytic effect of LLC-EVs can be abrogated by PTHR knockdown and treatment with a neutralizing anti-PTHrP antibody. Together, these data show that LLC-EV-induced lipolysis is mediated by extracellular PTHrP. These findings suggest a novel mechanism of lipid droplet loss and identify a potential therapeutic strategy for cancer cachexia.

## Supplementary information

supplementary figure and their legends

## Data Availability

All data analyzed during this study are included in this published article.
