# Supplementary material for: Extracellular vesicles-released parathyroid hormone-related protein from Lewis lung carcinoma induces lipolysis and adipose tissue browning in cancer cachexia
Source: Cell Death Dis. 2021 Jan 28;12(1):134. doi: 10.1038/s41419-020-03382-0 (PMC7843996; doi:10.1038/s41419-020-03382-0)
Supplement: Supplementary file 1 — supplementary figure and their legends [file 41419_2020_3382_MOESM1_ESM.docx]

**Supplementary material**

**Supplementary Figures**

**Figure S1**


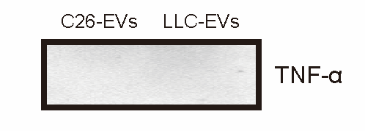


**Figure S1** The expression of TNF-α in LLC-EVs (50μg), C26-EVs (50μg).

**Figure S2**


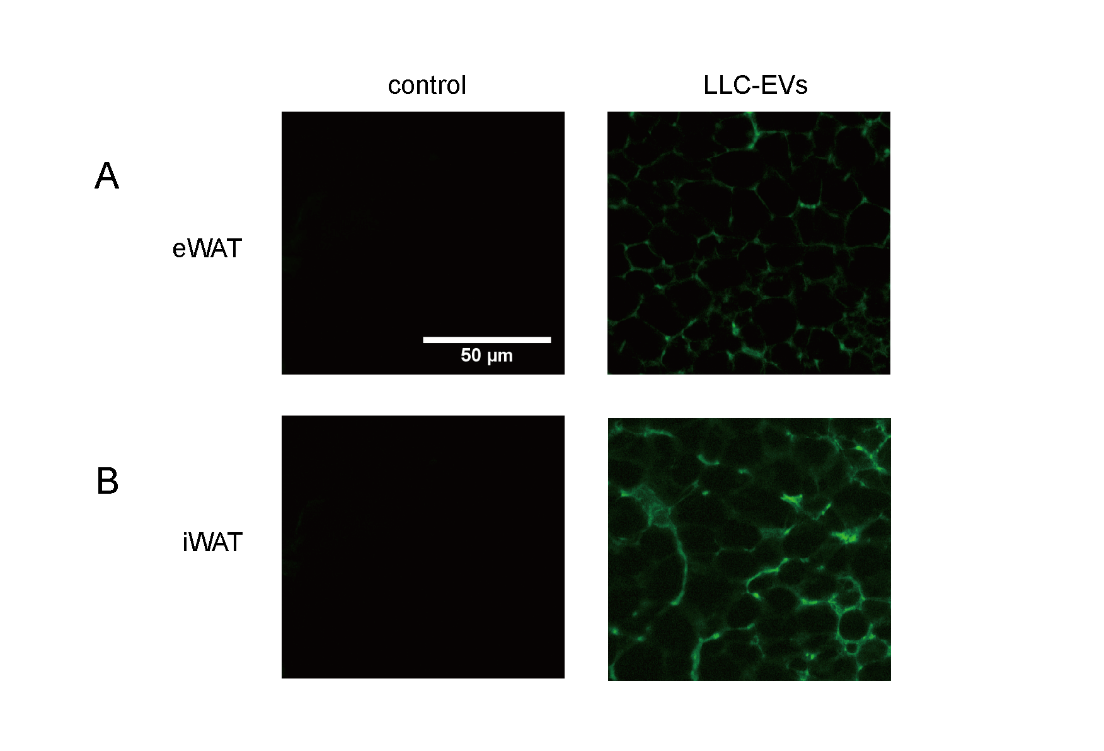


**Figure S2** LLC-derived EVs settle in subcutaneous and epididymal adipose tissue.

LLC-EVs labeled with PKH67 were administrated into tail veins of mice for 24h, fluorescence signals were detected in epididymal adipose tissue (A) and subcutaneous adipose tissue (B) by fluorescence microscope. Scale bar: 30μm.
